# A field study on the efficacy of ivermectin via subcutaneous route against chewing lice (*Bovicola caprae*) infestation in naturally infested goats

**DOI:** 10.4102/ojvr.v86i1.1635

**Published:** 2019-01-30

**Authors:** Y. Ajith, Umesh Dimri, A. Gopalakrishnan, Gopinath Devi

**Affiliations:** 1 Division of Medicine, ICAR-Indian Veterinary Research Institute, India; 2 Department of Veterinary Clinical Medicine, Tamil Nadu Veterinary and Animal Sciences University, India; 3 Department of Veterinary Medicine, ICAR-Indian Veterinary Research Institute, India

## Abstract

Caprine pediculosis is an ectoparasitic disease of great concern among goat farmers in India. It may be caused by either sucking lice or chewing lice; the latter one results in severe skin lesions, leading to production loss. This study evaluated the effectiveness of the macrocytic lactone drug, ivermectin, administered via subcutaneous injection, against chewing lice *Bovicola (Damalinia) caprae* infestation in naturally infested goats. The study was conducted on 20 goats with severe *B. caprae* infestation. Animals of group A (*n* = 10) were treated using a single dose of ivermectin (200 *µ*g/kg body weight) subcutaneously and animals of group B (*n* = 10) underwent placebo therapy using normal saline. The animals were examined on days 0, 3, 7, 14, 21, 28, 42 and 56 for lice counts. There was 100% elimination of lice in all animals of group A and effective protection from re-infection remained at least for 21 days. Considerable improvement in haematological parameters was also observed by day 21. Based on this study, ivermectin injected via a subcutaneous route can be used effectively for the therapeutic and prophylactic management of chewing lice infestation in goats maintained under an extensive grazing system.

## Introduction

Caprine pediculosis is considered as a major ectoparasitic condition commonly seen among goats reared under an extensive grazing system, mainly during the winter season (Iqbal et al. 2014). It is reported from almost all parts of the world, especially from the Dehradun valleys of the subtropical Himalayas, tropical Asia and parts of Africa (Adesh et al. 1994; Ajith et al. 2017a). Based on their feeding habits, aetiological agents of caprine pediculosis can be classified into two subgroups: sucking lice (suborder: Anoplura), which includes blood feeders such as *Linognathus stenopsis* and *L. africanus,* and chewing or biting lice (suborder: Mallophaga), which includes non-blood feeders such as *Bovicola (Damalinia) caprae* and *B. limbata* (Taylor, Coop & Wall 2007). The common chewing lice found among goats in India were *B. caprae* (Ajith et al. 2017a; Giri, Kashyap & Dewangan 2013). These are small (approximate size: 1 mm – 2 mm) permanently parasitic insects of the Phthiraptera family, seen close to the skin, and complete their whole life cycle on the host body surface and transmission occurs only by direct physical contact (Smith & Sherman 2009; Taylor et al. 2007). Agroclimatic region, breed, immune status, system of rearing and hygiene are the major factors affecting the prevalence and distribution of lice among goats (Ajith et al. 2017a). Alopecia, skin irritation, papulo-crustous dermatitis, self-excoriation, anaemia and stress-associated production loss and growth reduction were often found to be associated with Caprine pediculosis (Taylor et al. 2007). Chewing lice infestation induced T-helper cells-2 (Th2)-dominant immune response and conferred pro-oxidative haemato-biochemical damages in goats (Ajith et al. 2017b).

Successful control of chewing lice in goat herds depends on the method of application and efficacy of insecticide, which, in turn, depends on the distribution of insecticide on the body surface and redistribution to untreated parts. Failure in treatment may occur because of incorrect application, inability to reach effective licicidal concentration on skin surface and development of insecticidal resistance (Keys, Toothey & Arul 1993). Conventionally, topical insecticides such as amidines, organophosphates, carbamates and pyrethroids were used as dips or spray to control chewing lice infestation in animals. But low safety (potential for being toxic to the domestic animals), public health concerns (topical preparations can spread to animal handlers), practical difficulties (the distribution of insecticide depends on hair length and external application is not suitable because Caprine pediculosis is more prevalent during winter season) and risk of environmental contamination (goats are mainly reared under extensive rearing system, especially in India, so topically applied insecticide may get spread into the pasture) limited their therapeutic use in food animals, especially those reared under an extensive grazing system. Later, the pour-on formulation of synthetic pyrethroids became more popular and acceptable because of high efficiency, ease of application and rapid distribution on the body (McEwan Jenkinson et al. 1986). Flumethrin 1% pour-on (Bayticol^®^ Pour-On, 1% weight/volume [w/v]; Bayer, Germany) was reported to be 100% effective in the treatment of chewing lice (*B. caprae*) infestation in goats, preventing re-infestation for up to 42 days under experimental conditions (Garg, Katoch & Bhushan 1998). The possible demerit of that study design was the lack of possible routes of transmission, because chewing lice essentially need close contact or manual transfer of lice for re-infection. Pour-on treatments were reported to be less effective in goats because of the variability in hair fibre characteristics and lice occur more close towards skin surface (Taylor et al. 2007). Also, rapid development of resistance, high toxicity and concerns of residues in milk as well as meat and chance of environmental contamination discourage the use of topical pyrethroids in food animals, especially maintained under extensive system of rearing (Lakshmanan et al. 2013).

Macrocyclic lactones (avermectins and milbemycins) are a group of endo-ectoparasiticidal agents with a wide margin of safety and broad spectrum of activity. Injectable forms of agents such as ivermectin, doramectin and moxidectin were used against various ectoparasites and endoparasites, especially against haematophagous ectoparasites such as ticks, fleas and sucking lice, but not used clinically against chewing lice in goats because of lack of scientific evidence (Smith & Sherman 2009; Taylor et al. 2007). Ivermectin is an antiparasitic drug with a broad spectrum of activity, high efficacy and wide margin of safety. The pharmacokinetic study of ivermectin in goats showed that it persists in significant concentrations in skin and hair for a minimum of 17 days post-treatment after subcutaneous route of administration (Lespine et al. 2005). Even though topical preparations of macrocyclic lactones such as selamectin, eprinomectin, ivermectin and doramectin were found to be effective and giving persistent activity against biting lice infestation in dogs, cats, cattle and sheep, efficacy of subcutaneous injection of avermectins against chewing lice infestation is not yet investigated in goats (Holste et al. 1997; Lloyd et al. 2001; Rugg et al. 1995; Shanks et al. 2003). In view of this, a field study was designed to evaluate the effects of chewing lice on haematological parameters and the effectiveness of subcutaneous ivermectin in the management of chewing lice infestation among goats reared under extensive grazing system under natural conditions.

## Materials and methods

A herd of 24 black Bengal male goats kept for meat purpose, coming under the age group of 10–12 months with severe *B. caprae* infestation (cumulative count above 100 in 10 cm × 10 cm areas at six sites on the neck, shoulder, withers, inguinal, flank and rump), were included in the study. No animals used in the study population previously received any type of ectoparasiticidal therapy. Animals without any systemic signs of disease only were included. All animals of the herd were weighed and dewormed using oral albendazole suspension at the rate of 10 mg/kg body weight 3 weeks prior to the field study, and 20 animals showing no parasitic ova in faecal smear examination on day 0 of the clinical trial were randomly grouped into two groups: group A and group B.

Animals of group A (treatment group [*n* = 10]) were treated using ivermectin (Hitek injection, 1% w/v; Virbac India, Mumbai, India) on day 0 at the dose rate of 200 *µ*g/kg body weight subcutaneously. Animals of group B (*n* = 10) kept as control and were given a placebo therapy using 0.6 mL normal saline subcutaneously on day zero. Animals were kept under the same environmental and management conditions throughout the study and thus facilitated natural transmission of lice to the treatment group by constant exposure to infested untreated animals. All animals were allowed to graze for 8 h a day in the same grazing area, where green grass and fresh water were available. Animals were examined on days 0, 3, 7, 14, 21, 28, 42 and 56 for lice counts and severity of infestation was assessed by summation of total motile lice, counted using the standard counting technique (lice number was counted in 10 cm × 10 cm areas at each of the six sites on the neck, shoulder, withers, inguinal, flank and rump) (Higgs, Love & Morcombe 1994; Holdsworth et al. 2006). Percentage reduction was calculated by Garg’s formula and reduction efficacy was calculated using Abbott’s formula (Abbott 1925; Garg et al. 1998).

Garg’s formula:

percentage reduction=[1−(Ta/Ca×Cb/Tb)]×100, [Eqn 1]

Where,

*Ta* = infestation on treated animals after treatment

*Tb* = infestation on treated animals before treatment

*Ca* = infestation on control animal after treatment

*Cb* = infestation on control animals before treatment.

Abbott’s formula:

Reduction efficacy=100×[(C−T)/C], [Eqn 2]

Where,

*C* = parasite count of control group

*T* = parasite count of treatment group.

Blood samples were collected from the jugular vein of all animals of both groups on day 0 (before treatment) and day 21 to evaluate the alterations in haematological parameters such as haemoglobin (Hb), total erythrocytic count (TEC), total leukocytic count (TLC) and differential leukocytic count (DLC) by standard methods (Jain 1986).

### Ethical considerations

The study was conducted as per the World Association for the Advancement of Veterinary Parasitology (WAAVP) guidelines for evaluating the efficacy of ectoparasiticides against biting lice (Holdsworth et al. 2006). This work was approved under the project Department of Science and Technology – Sustainable Agriculture and Rural Transformation Holistic Initiative (DST-SARTHI) (SEED/SARTHI/HP/19/2012), funded by the Department of Science and Technology, Ministry of Science and Technology, Government of India, and was conducted in compliance with the universal ethical standards.

## Results

**FIGURE 1:**
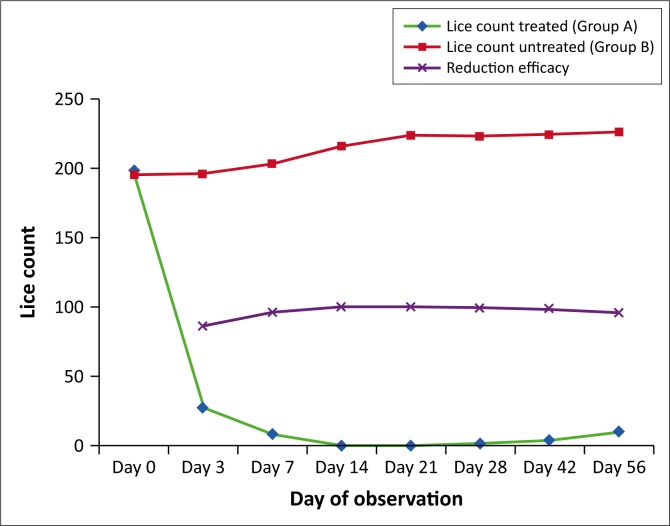
Effect of ivermectin (200 *µ*g/kg body weight via subcutaneous route) on mean number of lice (*Bovicola caprae*).

**FIGURE 2:**
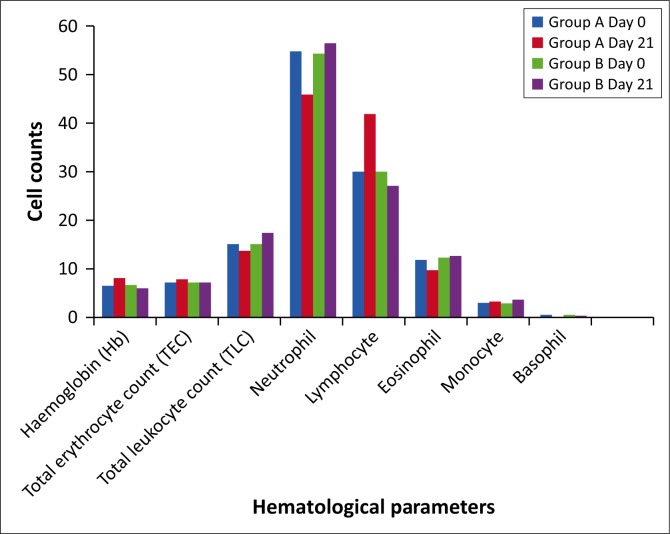
Alterations in haematological profile of lice (*Bovicola caprae*) infested goats before and after treatment (mean).

The mean number of *B. caprae* observed on each of the six sites during the study period was recorded (Table 1). Animals of group A showed a significant reduction in the number of lice by day 3 and complete elimination of lice population was found on day 14. Even though ivermectin-treated animals were in constant exposure to untreated lice-infested animals, they remained free from lice and lice eggs from day 14 to day 21 (Figure 1), which suggests that ivermectin via subcutaneous route could produce high licicidal concentration on skin surface at least for 21 days. In group A, two animals showed mild levels of lice infestation (cumulative count < 10 lice) on day 28 and all animals showed mild infection by day 56. There was a significant (*p* < 0.05) increase in lice count on animals of group B during the course of the study. Anaemia, leucocytosis, neutrophilia and eosinophilia were observed in chewing lice-infested animals before treatment and significant (*p* < 0.05) improvement towards normal values was noted in group A animals after treatment (Table 2 and Figure 2). No animals in the treatment group showed any adverse reactions.

**TABLE 1:** Mean lice (*Bovicola caprae*) count and intact lice egg (nits) in different body regions of goats.

Body region	Day 0	Day 3	Day 7	Day 14	Day 21	Day 28	Day 42	Day 56
**Group A – Ivermectin-treated group (*n* = 10)**			
Neck	54.3	9.6	4.3	0.0	0.0	0.7	1.7	4.3
Shoulder	43.8	7.2	1.4	0.0	0.0	0.5	1.1	3.1
Withers	32.2	3.2	0.9	0.0	0.0	0.2	0.8	1.2
Inguinal	21.3	4.1	1.2	0.0	0.0	0.0	0.2	0.5
Flank	24.9	1.9	0.3	0.0	0.0	0.0	0.0	0.4
Rump	21.1	1.2	0.1	0.0	0.0	0.0	0.0	0.1
Total count	197.6*	27.2*	8.2*	0.0*	0.0*	1.4*	3.8*	9.6*
Intact lice egg	Yes	Yes	No	No	No	No	No	Yes
Percentage reduction†	-	86.32	96.02	100.00	100.00	99.38	98.33	95.82
Reduction efficacy‡	-	86.13	95.97	100.00	100.00	99.37	98.31	95.76
**Group B – Control group (*n* = 10)**								
Neck	53.8	53.9	55.8	58.6	59.2	58.2	61.2	59.2
Shoulder	41.9	42.6	43.4	46.7	47.2	46.2	42.2	46.8
Withers	33.4	33.9	34.7	35.8	36.3	39.2	36.2	37.3
Inguinal	22.1	21.1	23.2	25.2	24.8	25.1	30.0	29.8
Flank	23.6	23.8	24.6	26.7	29.1	28.0	29.2	28.9
Rump	20.1	20.8	21.7	23.2	27.2	26.6	25.6	24.4
Total count	194.9**	196.1**	203.4**	216.2**	223.8**	223.3**	224.4**	226.4**
Intact lice egg	Yes	Yes	Yes	Yes	Yes	Yes	Yes	Yes

* , values differ significantly (*p* < 0.01) in a row;

** , values differ significantly (*p* < 0.05) in a row.

† , Calculated by Abbott’s formula – refer to Eqn 2.

‡ , Calculated by Abbott’s formula – refer to Eqn 1.

**TABLE 2:** Alterations in haematological profile of lice-infested goats before and after treatment (mean ± standard error).

Parameters	Unit	Group A (*n* = 10)		Group B (*n* = 10)		Reference range
		Day 0	Day 21	Day 0	Day 21	
Haemoglobin (Hb)	g/dL	6.48 ± 0.155**	8.14 ± 0.108***	6.64 ± 0.166**	5.98 ± 0.113***	8–12
Total erythrocyte count (TEC)	× 10^6^ cells/*µ*L	7.196 ± 0.06**	7.788 ± 0.053**	7.178 ± 0.051	7.06 ± 0.042	8–18
Total leukocyte count (TLC)	× 10^3^ cells/*µ*L	15.14 ± 0.282**	13.73 ± 0.226***	15.22 ± 0.186**	17.32 ± 0.266***	4–13
Differential leukocyte count (DLC)						
Neutrophil	%	54.8 ± 0.742**	45.1 ± 1.206***	54.2 ± 0.941	56.4 ± 0.921*	30–48
Lymphocyte	%	29.9 ± 0.936**	41.9 ± 1.456***	30.1 ± 0.767**	27.0 ± 1.247***	50–70
Eosinophil	%	11.8 ± 0.512**	9.7 ± 0.367***	12.3 ± 0.423	12.7 ± 0.448*	1–8
Monocyte	%	3.1 ± 0.314	3.2 ± 0.327	2.9 ± 0.277**	3.6 ± 0.267**	0–4
Basophil	%	0.4 ± 0.163**	0.1 ± 0.1***	0.5 ± 0.167	0.3 ± 0.153*	0–1

Note: Significant anaemia observed in goats with chewing lice infestation; subcutaneous ivermectin produced licicidal concentration on skin surfaces in chewing lice-infested goats; a single dose of ivermectin effectively eliminated chewing lice infestation for a minimum of 21 days.

* , values differ significantly (*p* < 0.05) in a row between group;

** , values differ significantly (*p* < 0.05) in a row within group between days;

*** , values differ significantly (*p* < 0.05) in a row between group and values differ significantly (*p* < 0.05) in a row within group between days

## Discussion and conclusion

In this study, the use of ivermectin via subcutaneous route was found effective in controlling chewing lice infestation on goats under field conditions. Treatment of caprine pediculosis had not been studied extensively, and the studies conducted on sheep and cattle were usually extrapolated for deciding therapeutic strategy in goats (Constable et al. 2016). The previous literature on the efficacy of macrocyclic lactones, especially ivermectin via subcutaneous route, in biting or chewing lice infestation in animals remained variable. Some earlier studies reported that injectable avermectins were not effective, even when topical pour-on formulations showed high efficacy in the same animal herd (Chick et al. 1993; Cleale et al. 2004; Titchener, Parry & Grimshaw 1994). Recently, several studies reported the beneficial and persistent effect of injectable avermectins in chewing lice infestation in ruminants. Morsy, Habib and Haridy (2001) observed complete cure from *B. caprae* infection in all goats parenterally treated with a combination of ivermectin and clorsulon by 1–2 weeks. Colwell (2002) reported that injectable moxidectin prevented re-infestation with *B. bovis* for up to 35 days. These variations in results may be attributed to the altered resistance pattern of the parasite and improved distribution, stability, persistent activity and pharmacokinetic properties of the injectable preparations of avermectins. Constable et al. (2016) appreciated these recent studies showing excellent efficacy and persistent effect by recommending both topical and injectable macrocyclic lactone-based products against both sucking and chewing lice in cattle.

The reports of resistance and tolerance towards conventional insecticides like pyrethroids in cattle and horse with chewing lice infestation are alarming (Ellse, Burden & Wall 2012; Sands et al. 2015). Also, these topical preparations are highly prone to environmental contamination and have a severe public health impact. Ivermectin is a highly lipophilic compound that dissolves in most of the organic solvents but is practically insoluble in water, which provides long duration of action and makes it least capable of environmental contamination. It has exceptional potency against endoparasites as well as ectoparasites at extremely low doses, which accounts for its wide margin of safety; hence, toxicity to ivermectin is very rare. The greatest bioavailability, longer duration of activity and better efficacy is achieved by subcutaneous route of administration when compared with the topical application (Canga et al. 2009). Recent reports on the better therapeutic efficacy of newer generation insecticides such as fipronil (phenylpyrazoles) and imidacloprid (neonicotinoids) in controlling chewing lice infestation of dogs and horses are worth outstanding (Kužner et al. 2013; Mencke et al. 2004). Some essential oils and oil-based formulations are also shown to have *in vitro* licicidal, as well as ovicidal, effect and are suggested as environment-friendly toxicologically safe alternative in controlling chewing lice infestation in donkey (Sands, Ellse & Wall 2016; Talbert & Wall 2012).

As with other parasitic diseases, antigens from the lice saliva might evoke strong humoral immune response, resulting in anaemia, mild neutrophilia and severe eosinophilia. In this study, significant level of anaemia, leucocytosis, neutrophilia and eosinophilia was observed in chewing lice-infested animals. Such alterations in haematological parameters were previously reported in calves and goats infested with chewing or biting lice or sucking lice (Ajith et al. 2017b; Devaney et al. 1992; Otter et al. 2003). Even though chewing or biting lice feed on epidermal exudates and debris from upper skin layers, the increased infestation of these parasites may lead to severe inflammatory reactions on skin surfaces. The resultant tissue scaling and inflammatory changes on skin surface cause oxidative stress and alter haemato-biochemical values in caprine pediculosis (Ajith et al. 2017b). Several vasodilators, anticoagulants, histamine binding proteins and immunosuppressive compounds are present in the saliva of human lice (Jones 1998). The possible role of these chemicals present in lice saliva in haematological changes needs to be explored in the future studies. The significant improvement in the haematological parameters on day 21 of ivermectin therapy can be attributed to the recovery from lice-induced damages. In lice-infested goats, the significant decrease in serum mineral, calcium, phosphorus, copper and zinc, levels was also reported earlier (Dede, Deger & Deger 2003).

The current study showed complete clearance of lice infestation in all animals by day 14 after subcutaneous ivermectin therapy and the treated animals remained uninfected from day 14 to day 21, even when the maximum chance for natural transmission from infested untreated animals was provided. Even after day 21, the lice counts remained significantly low till day 56 of therapy, which makes clear that ivermectin has persistent licicidal activity and long half-life after subcutaneous administration in goats and is efficient in controlling chewing lice. Hence, it can be concluded that injectable ivermectin can maintain licicidal concentrations on the skin surface and is 100% effective in controlling chewing lice (*B. caprae*) infestations for a minimum of 21 days.
